# Evolutionary Analysis of Dipeptidyl Peptidase I

**DOI:** 10.3390/ijms23031852

**Published:** 2022-02-06

**Authors:** Nina Varda, Marko Novinec

**Affiliations:** Department of Chemistry and Biochemistry, Faculty of Chemistry and Chemical Technology, University of Ljubljana, Večna pot 113, 1000 Ljubljana, Slovenia; nv7054@student.uni-lj.si

**Keywords:** oligomerization, molecular evolution, cathepsin C

## Abstract

Human dipeptidyl peptidase I (DPPI) belongs to the family of papain-like cysteine peptidases. Its distinctive features are the unique exclusion domain which enables the eponymous activity and homotetramerization of DPPI, and its dependence on chloride ions for enzymatic activity. The oligomeric state of DPPI is unique in this family of predominantly monomeric peptidases. However, a distant DPPI ortholog from *Plasmodium falciparum* has been shown to be monomeric, indicating that the oligomeric state of DPPI varies between lineages. The aim of this work was to study the evolution of DPPI, with particular attention to the structural features that determine its characteristic enzymatic activity and preferences, and to reconstruct the evolution of its oligomerization. We analyzed fifty-seven selected sequences of DPPI and confirmed its presence in three lineages, namely, Amorphea (including animals and Amoebozoa), Alveolates and the metamonad *Giardia*. The amino acid residues that bind the chloride ion are highly conserved in all species, indicating that the dependence on chloride ions for activity is an evolutionarily conserved feature of DPPI. The number of N-glycosylation sites is significantly increased in animals, particularly vertebrates. Analysis of homology models and subunit contacts suggests that oligomerization is likely restricted to DPPIs in the Amorphea group.

## 1. Introduction

Dipeptidyl peptidase I (DPPI; EC number 3.4.14.1), also known as cathepsin C, is a cysteine peptidase located in the lysosome. It belongs to the family of papain-like cysteine peptidases [1] and is classified in the C01.070 group in the MEROPS peptidase database [2]. It plays an important role in protein degradation and activation of enzymes, especially serine peptidases in immune cells [3]. Mutations in DPPI that cause its insufficient activity highlight its biological importance, as they can lead to diseases such as Papillon–Lefevre and Haim–Munk syndromes [4]. On the other hand, inhibition of DPPI is a promising strategy for the treatment of inflammatory diseases [5] and cancer [6].

**Figure 1:**
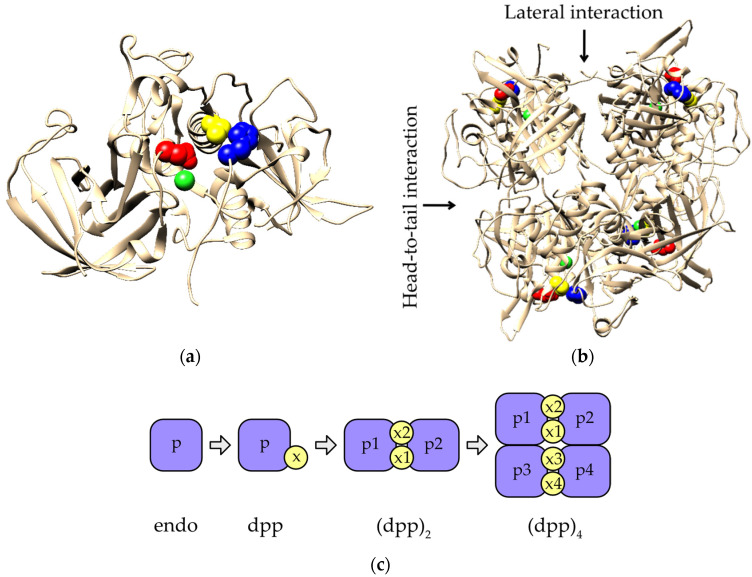
(**a**) Crystal structure of a single subunit of human DPPI (PDB accession code 1k3b). The protein is shown in cartoon representation. The residue Asp1 (red) and the catalytic diad of Cys234 (yellow) and His381 (blue) are shown as spheres. The bound chloride ion is shown as a green sphere. (**b**) Homotetrameric form of human DPPI. Head-to-tail and lateral interactions between subunits are indicated by arrows. Visualization colors correspond to those in panel a. (**c**) Stepwise evolution of DPPI. Addition of the exclusion domain (x) to the peptidase domain (p) of an endopeptidase ancestor (endo) gave rise to a dipeptidyl peptidase (dpp). Later, dpp associated to form homotetramers via an intermediate dimeric form (dpp_2_), found at the zymogen level in mammals.

DPPI is unique in its family because, unlike other members that are monomers, mature mammalian DPPI is a homotetramer (Figure 1a,b). In addition to its catalytic domain, which consists of heavy and light chains, it also contains an N-terminal exclusion domain that has a fold similar to that of metalloprotease inhibitors. It contributes significantly to subunit interactions and is therefore thought to be responsible for tetramerization [7]. The exclusion domain is non-covalently bound to the catalytic domain in an orientation that prevents substrate binding beyond subsite S2 and gives the enzyme its namesake activity. This is further supported by the N-terminal residue Asp1 of the exclusion domain, which stabilizes the N-terminus of the substrate [7,8]. It has been shown that the exclusion domain is not necessary for the activity of the enzyme. However, its removal resulted in a monomeric endopeptidase, confirming its importance for tetramerization [9]. In addition to the exclusion domain, proper N-glycosylation has also been shown to play a crucial role in this process in mammals [10,11], even though de-glycosylated bovine DPPI retains most of its activity [12]. This was confirmed by our finding that recombinant human DPPI produced in *E. coli* is a fully functional monomer [11]. Nevertheless, mature mammalian DPPI is a dimer of dimers that is sequentially assembled via a dimeric form present at the level of the pro-DPPI zymogen. Because of this specific geometrical arrangement, there are two distinct interaction surfaces between the subunits (Figure 1b). In agreement with the published literature [7,13], we refer to them as head-to-tail and lateral interactions. Based on the crystal structure of human DPPI, Turk and coworkers proposed that head-to-tail dimers are formed first [7]. In contrast, Lamort and coworkers recently investigated the processing and assembly of human DPPI using experimental and computational methods and argued that pro-DPPI consists of a lateral dimer [13]. It was also shown that the residue Cys331 plays an important role in tetramer formation [12]. Another characteristic feature of DPPI at the active enzyme level is the binding of a chloride ion in the S2 binding pocket, which is necessary for enzyme activity [7].

Distant DPPI orthologs have been identified in *Plasmodium falciparum* [14] that exhibit conserved dipeptidyl peptidase activity and have been designated dipeptidyl aminopeptidases (DPAPs). Of the three DPAPs encoded in the *Plasmodium falciparum* genome, two (DPAP1 and DPAP3) are associated with the intra-erythrocytic part of the plasmodial life cycle that causes the typical symptoms of malaria [14,15]. Interestingly, its biochemical characterization has shown that DPAP1 is a monomer [16]. Similarly, three DPPI homologs (CPCs) have been identified in *Toxoplasma gondii* and shown to be important for the intracellular survival of this parasite [17]. Taken together, the known data suggest that DPPI evolved in a stepwise manner, as shown in Figure 1c, starting with the fusion of the exclusion domain with a papain-like peptidase that gave rise to a functional dipeptidyl peptidase, followed by association with dimeric and tetrameric forms. In this article, we aim to gain further insight into the evolutionary history of DPPI by examining its diversity and conservation, focusing on its characteristic structural and functional elements.

## 2. Results

### 2.1. Phylogenetic Analysis

**Figure 2:**
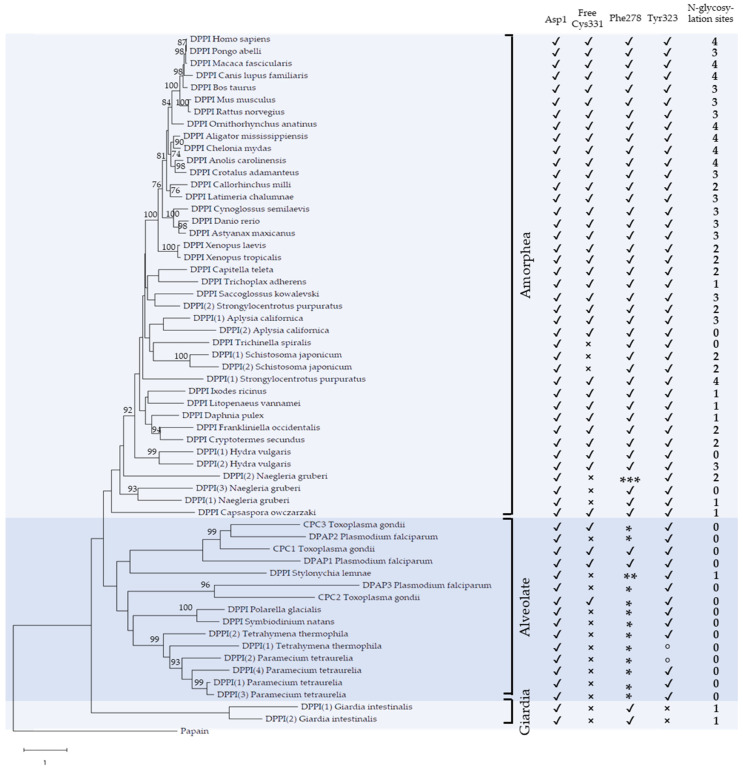
Maximum likelihood phylogenetic tree of DPPI. The tree was rooted using the sequence of papain from *Carica papaya* as an outgroup. Only bootstrap values above 75 are shown. The table on the right highlights the conservation of characteristic features of human DPPI, i.e., Asp 1, free Cys331, Cl^-^ binding residues in the S2 binding pocket (Phe278, Tyr280) and the number of predicted N-glycosylation sites (**✓**—conserved; ×—not conserved; *—Tyr is present instead of Phe; **—Ile is present instead of Phe; ***—Trp is present instead of Phe; °—Phe is present instead of Tyr).

Fifty-seven complete sequences of DPPI were collected from publicly available databases (Supplementary Table S1) and aligned (Supplementary Dataset S1). The range of organisms included a selection of representative animal species as well as unicellular eukaryotes and, to the best of our knowledge, is representative of the phylogenetic distribution of DPPI. The evolutionary relationships among the included sequences are shown in the maximum likelihood phylogenetic tree in Figure 2. A single DPPI was found in vertebrates, but lineage-specific duplications were observed in several invertebrate animals and, more frequently, in unicellular eukaryotes, where up to four paralogs were identified. In addition, Figure 2 summarizes the conservation of specific structural elements which we will analyze in more detail in the continuation of this article. These include Asp1 involved in substrate binding [7], Phe278 and Tyr323 involved in chloride ion binding in the S2 binding pocket [7], free Cys331 important for tetramer formation [12] and the number of N-glycosylation sites predicted based on the consensus sequence Asn-X-Ser/Thr (all residues throughout the manuscript are numbered according to the numbering of human pro-DPPI).

The evolutionary groups of DPPI observed in the phylogenetic tree correspond, for the most part, to the evolutionary relationships between the taxonomic groups. On this basis, the tree can be divided into three groups—Amorphea, Alveolates and the metamonad *Giardia intestinalis*. Within the Amorphea group, which contains animals, Amoebozoa (*Naegleria fowleri*) and *Capsaspora owczarzaki*, vertebrate DPPIs are separated from the rest of the lineages with high bootstrap support, while relationships between other DPPIs are less well supported. Most species contain a single DPPI, but in some cases, duplications are observed, resulting in two paralogs in certain invertebrates (*Aplysia californica, Hydra vulgaris, Schistosoma mansoni* and *Strongylocentrotus purpuratus*) and three paralogs in the amoeba *Naegleria fowleri*. The Alveolate group contains all DPPI sequences from species in the namesake lineage. It includes DPPIs from *Plasmodium falciparum* and *Toxoplasma gondii*, for which we retain the designations DPAPs and CPCs known from the published literature [14,17], as well as DPPIs from ciliates (*Stylonichia lemnae, Tetrahymena thermophila* and *Paramecium tetraurelia*) and dinoflagellates (*Polarella glacialis* and *Symbiodinium natans*). In the MEROPS database, *Plasmodium* DPAPs are classified separately from animal DPPIs and are divided into DPAP1 (C01.124) and DPAP3 (C01.139) [2]. The phylogenetic analysis in Figure 2 reflects this division, as DPAP1 and DPAP2 are separated from DPAP3, while a third subgroup is formed by DPPIs from ciliates and dinoflagellates. This analysis also sheds light on the evolutionary relationships between DPAPs and CPCs. To investigate the functional relationships between the Alveolate and Amorphea groups, we analyzed their functional divergence using the software DIVERGE [18]. We found that type I, but not type II, functional divergence between the two groups was statistically significant (coefficient of functional divergence θ = 0.29 ± 0.02) [19,20]. Examination of the residues responsible for the divergence revealed that it can be explained by the presence of conserved residues in the Amorphea group, which are replaced by variable residues in the Alveolate group. The functional divergence between the two groups is not surprising considering that specialized roles of these enzymes have been described in *Plasmodium falciparum* and *Toxoplasma gondii* [14,15,17].

Significant divergence between the main groups as well as within the Alveolate group is also indicated by the long branch lengths in the tree. Consequently, the positions of a substantial number of branches in the tree are not well supported by bootstrap values. Nevertheless, the phylogenetic tree provides an adequate basis for further analysis of the characteristic DPPI features.

### 2.2. Primary and Tertiary Structure Conservation

Sequence conservation of individual residues in the mature DPPI is shown in Supplementary Figure S1. Overall, the catalytic domain is more conserved than the exclusion domain. The latter shows good conservation of the interface with the catalytic domain within the monomeric unit but significantly less in other parts of the molecule, including those involved in interaction with neighboring subunits in human DPPI.

**Figure 3:**
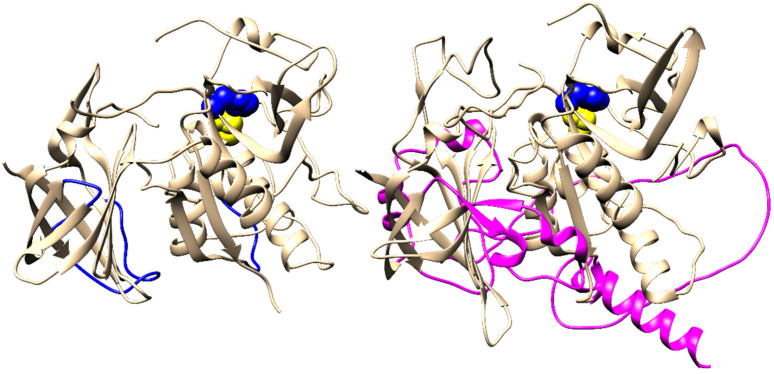
Structural comparison of Amorphea and Alveolate DPPI. The crystal structure of a single subunit of human DPPI is shown on the left (PDB accession code 1k3b), and the homology model of *Plasmodium falciparum* DPAPI built by AlphaFold [21] is shown on the right. Regions containing insertions of significant length in the latter are highlighted in blue and magenta, respectively. The catalytic diad Cys–His is shown as yellow and blue spheres.

In terms of tertiary structure conservation, Alveolate DPPIs contain several insertions of considerable size compared with Amorphea DPPIs. This is shown in Figure 3, which shows a comparison between the fold of human DPPI and the structural model of DPAP1 from *P. falciparum* generated with AlphaFold (Supplementary Dataset S2) [21]. The superposition of DPAP1 with the human DPPI tetramer shows that these insertions would also sterically hinder the formation of lateral and head-to-tail interactions between the subunits (Supplementary Figure S2), thus preventing the oligomerization of DPAP1 via the same geometric arrangement of subunits as in human DPPI. This is consistent with experimental results showing that DPAP1 is a monomer [16].

Conversely, the sequence length differs only slightly in the Amorphea group, with the greatest diversity observed in the propeptide region. Within the mature region, significant variation in the sequence length is rare. Most notable are the insertions of a few residues that occur in the DPPIs of *Ixodes icinus* and *Naegleria gruberi* (see multiple sequence alignment provided in Supplementary Dataset S1). However, molecular modeling shows that these insertions do not significantly affect the fold of these DPPIs (Supplementary Figure S3) and would not interfere with the oligomerization of the proteins assuming the same subunit arrangement as in human DPPI.

### 2.3. Conservation of Functionally Important Structural Elements

In the continuation of this study, we investigated the conservation of characteristic structural elements known to be important for the structure and function of DPPI in mammals. The catalytic diad Cys234–His381 as well as residues Gln228, Asn403 and Trp405, which are important for the catalytic activity of papain-like peptidases [1], are conserved in all sequences, indicating that they are all active peptidases. The same is true for Asp1, which stabilizes the N-terminus of the substrate [7], indicating that all enzymes also have conserved dipeptidyl peptidase activity.

A characteristic functional feature of mammalian DPPI is its dependence on chloride ions for activity [22,23], and this requirement is also conserved in DPAP1 of *P. falciparum* [16]. The crystal structure of human DPPI shows that a chloride ion is bound deep in the S2 binding pocket of each subunit, surrounded by several hydrophobic residues (Supplementary Figure S4a). It is partially obscured by Phe278 and stabilized by hydrogen bonding with the hydroxyl group of Tyr323 [7]. Phe278 is absolutely conserved in the Amorphea group, while Tyr323 is absent in only one case (Figure 2). In the Alveolate group, Phe278 is typically replaced by Tyr, while Tyr323 is conserved except for two cases in which it is replaced by Phe. In *Giardia intestinalis*, Phe278 is conserved, but Tyr323 is not. Taken together, the ability to bind chloride into the S2 pocket is a widely conserved feature of DPPIs and reinforces the prediction that most, if not all, of these enzymes have conserved dipeptidyl peptidase activity similar to that of mammalian DPPIs and *Plasmodium* DPAPs.

The free Cys residue at position 331, located at the lateral dimer interface (Supplementary Figure S4b), has been shown to be crucial for the tetramerization of bovine DPPI [12]. This residue is highly conserved in Amorphea DPPIs and is absent only in *Schistosoma mansoni*, *Trichinella spiralis* and *Naegleria gruberi*. In five of the six proteins in question, Cys is replaced by Ser or Thr, both of which have a similar size and chemical properties. It is unlikely that these substitutions have a significant effect on protein structure or function. In contrast, Cys is present at this position in only a few Alveolate DPPIs and is usually replaced by a residue of similar size (Ser, Thr, Ala, Val; see the multiple sequence alignment in Supplementary Dataset S1 for more details).

Human DPPI contains four N-glycosylation sites in each subunit: three in the exclusion domain (Asn5, Asn29 and Asn95), and the fourth (Asn252) at the bottom of the catalytic domain (Supplementary Figure S4c). Based on the consensus sequence for N-glycosylation, we examined the remaining sequences in the alignment. As shown in Figure 2, the number of N-glycosylation sites is highest in the Amorphea group (up to four), with a statistically significant increase in vertebrates compared to invertebrates, as confirmed by the Kruskal–Wallis test (*p* < 0.01). Invertebrate DPPIs exhibit high diversity in the number of N-glycosylation sites (even between paralogs), ranging from none to four, whereas all vertebrate DPPIs contain two to four such sites. In *Giardia intestinalis*, one N-glycosylation site is present in each paralog, whereas most Alveolate DPPIs have no N-glycosylation sites at all. Statistically significant differences were confirmed between invertebrates and Alveolates (*p* < 0.01), but not between *Giardia intestinalis* and Alveolates.

### 2.4. Evolution of Subunit Interfaces

**Figure 4:**
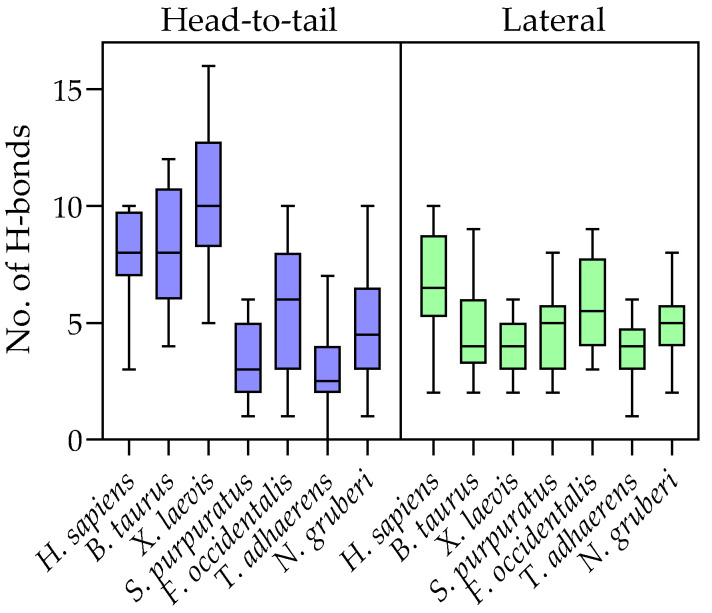
Conservation of interaction surfaces in DPPI examined by statistical analysis of hydrogen bonds between subunits in ten models of DPPI tetramers from selected species. Error bars denote the minimal and maximal values determined for each homolog. The plot was drawn using GraphPad Prism 9.3 software.

The human DPPI tetramer is constructed as a dimer of dimers, and it has been proposed that it assembles stepwise via a dimeric intermediate form at the proenzyme stage. There are two distinct sets of isologous subunit interfaces resulting from head-to-tail and lateral interactions (see Figure 1b). The head-to-tail interaction involves a buried surface area (BSA) of about 1100 Å^2^, and the lateral interaction involves a BSA of about 700 Å^2^. Statistically, protein–protein interaction surfaces of similar size contain, on average, four to six hydrogen bonds [24]. In the analyzed crystal structure of human DPPI (PDB accession code 1k3b), ten hydrogen bonds were detected in the head-to-tail interaction and twelve in the lateral interaction. In an effort to predict the oligomeric states of Amorphea DPPIs from divergent lineages, we constructed homology models of the putative tetrameric forms of six orthologs covering the diversity of DPPIs. For this task, Modeller [25] was chosen over AlphaFold because it allowed the generation of multiple models with four subunits, each containing three polypeptide chains. We included orthologs from cattle (*Bos taurus*), frogs (*Xenopus laevis*), echinoderms (DPPI(1) from *Strongylocentrotus purpuratus*), insects (*Frankliniella occidentalis*), placozoans (*Trichoplax adhaerens*) and amoebae (DPPI(1) from *Naegleria gruberi*). All models are provided in Supplementary Dataset S2. We determined the number of hydrogen bonds between subunits for ten generated models of each DPPI homolog. To assess the quality of the results, we also generated models of human DPPI and analyzed the number of hydrogen bonds therein. The results in Figure 4 show that, on average, human DPPI models contained fewer hydrogen bonds between subunits than the crystal structure, and that the upper limit of modeled hydrogen bonds was comparable to the crystal structure. Analysis of homology models from other species showed that the average and maximum numbers of hydrogen bonds involved in head-to-tail interactions are higher in vertebrates (cattle and frog) than in invertebrates and amoebae, whereas the differences in the number of hydrogen bonds involved in lateral interactions are less pronounced. Importantly, the average and maximum numbers of hydrogen bonds observed in all interaction surfaces indicate that these interactions would be stable, if formed. Thus, the models support the formation of oligomeric structures across both interfaces in Amorphea DPPIs.

**Figure 5:**
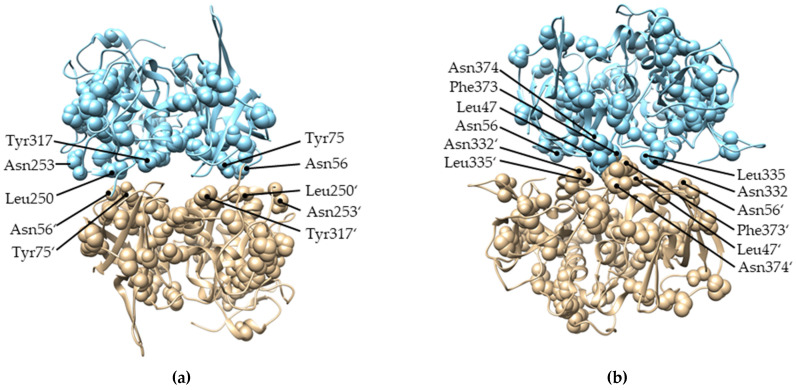
Residues responsible for type I functional divergence between Amorphea and Alveolate DPPIs mapped on the structure of human DPPI (PDB accession code 1k3b). For clarity of presentation, two subunits of DPPI are shown in a (**a**) head-to-tail interaction and (**b**) lateral interaction. Apostrophes are used to distinguish residues from different subunits.

We also examined the functional divergence between Alveolate and Amorphea DPPIs in the context of DPPI tetramerization. As mentioned above, type I functional divergence was observed between these two major DPPI lineages. Mapping of the residues responsible for the functional divergence on the structure of DPPI shows that, although they are distributed throughout the protein (listed in Supplementary Table S2), a considerable number of them are located in the interaction surfaces between the subunits (Figure 5). In the continuation of this study, we also examined the functional divergence between different lineages within the Amorphea group. No statistically significant divergence was detected between animal and non-animal DPPIs (i.e., *Naegleria fowleri* and *Capsaspora owczarzaki*), whereas type I functional divergence was detected between vertebrates and invertebrates. However, the coefficient of functional divergence was low (**θ** = 0.14 ± 0.03), and posterior probability values were above the threshold (*p* > 0.5) for only three residues. Taken together, these data suggest that there is no significant functional divergence among Amorphea DPPIs.

## 3. Discussion

Human DPPI is an attractive protein from both medicinal and scientific perspectives. In this article, we examined its evolutionary conservation and diversity. Its presence in animals and divergent unicellular eukaryotes (amoebae, Alveolates, metamonads) and its absence in prokaryotes suggest its origin at an early stage of eukaryote evolution, but due to the still missing relationships between eukaryote groups [26], its exact time of origin remains unclear. It should be noted that DPPI is, by no means, ubiquitous in eukaryotes, as it is absent in some major evolutionary groups such as plants and fungi, as discussed in [1,27]. For example, it is also absent in trypanosomes, which are otherwise well known for their papain-like and other peptidases [28].

Our analysis highlights the divergent evolution of DPPI between Amorphea and Alveolates, and further diversification in the latter lineage, which also has, on average, the most DPPI paralogs per species. Unfortunately, the functional properties and physiological roles of many of these enzymes remain largely unknown. Data are available mainly on DPPI in mammals, where this enzyme is ubiquitously expressed and performs numerous physiological and pathological functions [3,4,5], as well as on a few parasitic Alveolates. In *Plasmodium falciparum*, DPAPs contribute to erythrocyte invasion [14,15], and their counterparts in *Toxoplasma gondii* appear to perform similar functions [29].

Notwithstanding their different physiological roles, mammalian DPPIs and *Plasmodium* DPAPs have similar substrate specificity [30] and a conserved requirement for chloride ions for enzymatic activity [16], which is identified here as a universally conserved functional property of all DPPIs. The main difference between these enzymes is that *Plasmodium* DPAPs appear to be monomers [16], whereas mammalian DPPIs are homotetramers. Most papain-like peptidases are monomers, but there are also some oligomeric members of the family, including the distantly related homohexameric bleomycin hydrolase [31] and cathepsin X, which was recently shown to form a homodimer [32]. While the ability to form homotetramers depends on the exclusion domain of DPPI [7], other factors also appear to play a role. We recently described a recombinant form of DPPI produced in *E. coli* that was an active monomer [11]. A major difference between *E. coli*-expressed and native human DPPI is the absence of N-glycosylation in the former. It has been established that at least partial N-glycosylation of the exclusion domain is a prerequisite for proper maturation of rat cathepsin C in the cell [10], but removal of N-linked glycans from the mature form did not significantly affect the activity of bovine or human DPPI [11,12]. The evolutionary history of DPPI shows a variation in N-glycosylation consensus sequences from zero to four throughout the animal kingdom, with three or four typically found in vertebrates (Figure 2). Homology modeling of putative tetrameric forms of various DPPIs from Amorphea showed that, in all cases, sufficient hydrogen bonds can be formed between the subunits to allow oligomer formation (Figure 4). This is also supported by the analysis of functional divergence between Amorphea and Alveolate DPPIs which indicates the occurrence of conserved residues in subunit interfaces in the former lineage. Taken together, these data suggest that oligomerization is a conserved property of Amorphea DPPIs and that it is not generally dependent on a particular N-glycosylation pattern. The same is true for the presence of Cys331, which has been shown to be important for tetramer formation in bovine DPPI [12]. However, it should be noted that the actual oligomeric states and quaternary structures of these DPPIs, whether monomeric, dimeric or tetrameric, need to be verified experimentally to obtain definitive answers.

Finally, it should be noted that some specific structural features of DPPI cannot be addressed by computational methods alone. One of them is the proteolytic processing of the precursor leading to a mature enzyme consisting of three polypeptide chains per subunit, i.e., the exclusion domain and the heavy and light chains of the catalytic domain. This characteristic pattern is conserved between mammalian DPPIs [12,33] and *Plasmodium* DPAPs [16], and thus we can assume that it is also conserved in other homologs. However, to be certain, this assumption would need to be confirmed experimentally. From this point of view, this manuscript provides an excellent basis for future studies of the biochemical and physiological properties of DPPI enzymes.

## 4. Materials and Methods

### 4.1. Sequence Retrieval

Complete amino acid sequences of DPPIs from different organisms were collected from publicly available databases. The basic criterion was that the sequences were complete and included all segments, i.e., the N-terminal signal peptide, exclusion domain, propeptide and catalytic domain, as well as the conserved catalytic diad Cys–His. Only sequences with >90% coverage relative to the human DPPI sequence were included. As the primary source of data, we used the *MEROPS* database [2] where DPPI is classified under the ID C01.070. Additional sequences were collected from the Uniprot and NCBI databases. The final list of fifty-eight sequences selected for phylogenetic analysis and their accession numbers are shown in Supplementary Table S1.

### 4.2. Phylogenetic Analysis

MEGA X was used for the phylogenetic analysis [34]. The sequences were aligned using the ClustalW algorithm (Supplementary File S1) and additionally refined with PROMALS3D [35]. The adequacy of the alignment was verified manually with the alignment of Asp1 residues and the active site residues. The phylogenetic tree was inferred by using the maximum likelihood method and the LG substitution model [36]. A discrete Gamma distribution with 5 categories was used to model evolutionary rate differences among sites. To determine the reliability of the tree, bootstrapping was used with 500 replications. Conservation of specific amino acid residues, specified in the text, was determined by manual examination of the alignment.

### 4.3. Functional Divergence Analysis

Functional divergence was analyzed using DIVERGE 3.0 software [18]. The multiple sequence alignment (Supplementary File S1) and maximum likelihood tree (Figure 2) constructed as described in the previous section were used as input for the analysis. Both type I and type II functional divergences were determined between clusters representing different evolutionary groups (Amorphea, Alveolates, animals, vertebrates, invertebrates) [19,20]. Type I functional divergence was determined by both available options with similar results. The presented results were obtained by the “Gu99” method [19].

### 4.4. Three-Dimensional Representations and Structural Analyses

All images of three-dimensional representations of protein structures were produced in UCSF Chimera [37]. The structure of mature human DPPI was visualized using coordinates deposited in the RCSB Protein Data Bank (www.rcsb.org) under accession number 1k3b [7]. Three-dimensional models of other shown proteins were generated, as described in the following sections. Sequence conservation was mapped to the structure of human DPPI using the generated multiple sequence alignment (Supplementary File S1) and the built-in “Multialign Viewer” and “Render by Attribute” functions. Hydrogen bonds were detected with the “FindHBonds” function using relaxed constraints with default parameters.

### 4.5. Homology Modeling with AlphaFold

The structure of *Plasmodium falciparum* DPAP1 was built using Alphafold [21] available on Google Colab (Google LLC, Mountain View, CA, USA). The sequence was manually edited to remove the propeptide and to include the break between the light and heavy chains of the catalytic domain (based on the alignment with human DPPI) prior to its upload onto the server.

### 4.6. Homology Modeling with Modeller

Homology models of putative tetrameric forms of six DPPI homologs (*B. taurus, X. laevis, S. purpuratus, F. occidentalis, T. adhaerens* and *N. gruberi*) were built using Modeller 10v1 [25] based on the alignment generated herein. The crystal structure of human DPPI (PDB accession code 1k3b) was used as the template. For each homolog, 10 models were generated with the “automodel” routine.

### 4.7. Statistical Analysis of N-Glycosylation Sites

Statistical analysis of the number of N-glycosylation sites in different evolutionary lineages was performed with GraphPad Prism 9.3 software (GraphPad Software, La Jolla, CA, USA) using a non-parametric Kruskal–Wallis test followed by Dunn’s multiple comparisons test.

## Data Availability

Data are contained within the article or Supplementary Material.

## Supplementary Materials

The following are available online at https://www.mdpi.com/article/10.3390/ijms23031852/s1.
