# Supplementary material for: Evolutionary Analysis of Dipeptidyl Peptidase I
Source: Int J Mol Sci. 2022 Feb 6;23(3):1852. doi: 10.3390/ijms23031852 (PMC8836896; doi:10.3390/ijms23031852)
Supplement: Supplementary file 1 [file ijms-23-01852-s001.zip › Supplementary Figures and Tables.pdf]

Evolutionary analysis of dipeptidyl peptidase I

Nina Varda and Marko Novinec

University of Ljubljana, Faculty of Chemistry and Chemical Technology, Department of Chemistry and Biochemistry, Večna pot 113, 1000 Ljubljana, Slovenia

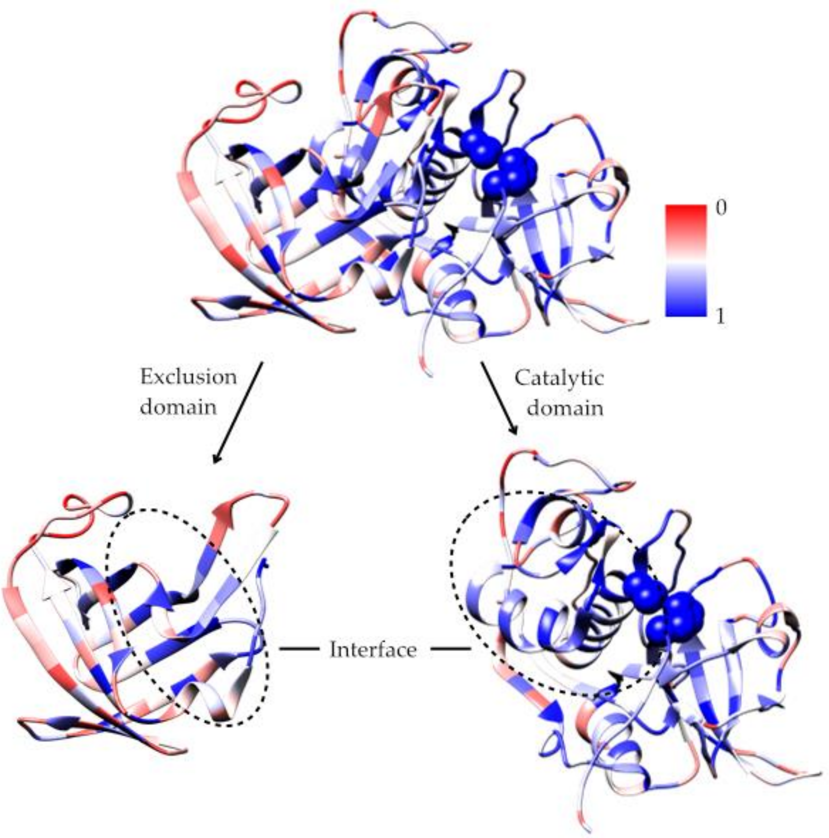

Supplementary Figure S1. Residue conservation mapped on the structure of human DPPI (PDB accession code 1k3b). The color code corresponds to residue conservation at individual positions calculated with the MultiAlign Viewer in UCSF Chimera. The interface between exclusion and catalytic domains is marked.

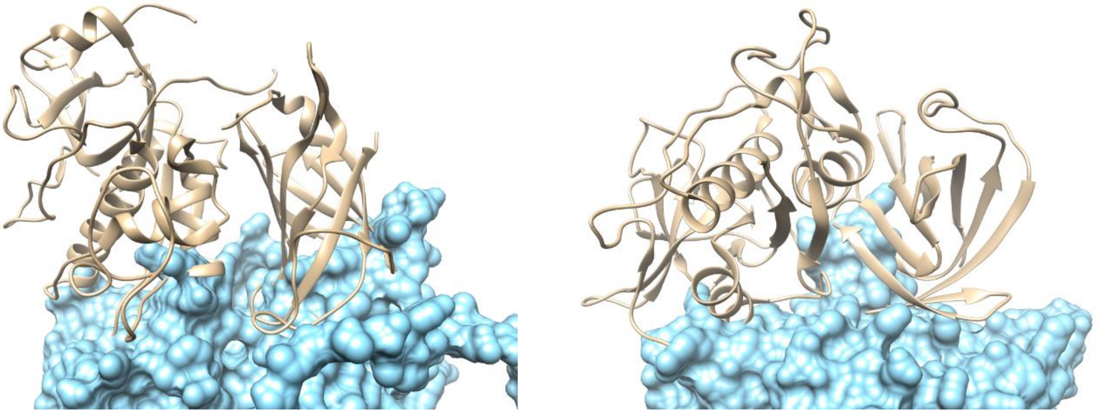

Supplementary Figure S2. Superposition of the homology model of *P. falciparum* DPAP1 on the human DPPI tetramer highlighting clashes with neighbouring subunits in head-to-tail (left) and lateral interactions (right). Human DPPI is shown in cartoon representation and DPAP1 in molecular surface representation. The crystal structure was retrieved from the Protein Data Bank under accession number 1k3b and the homology model was built with AlphaFold, as described in the main text.

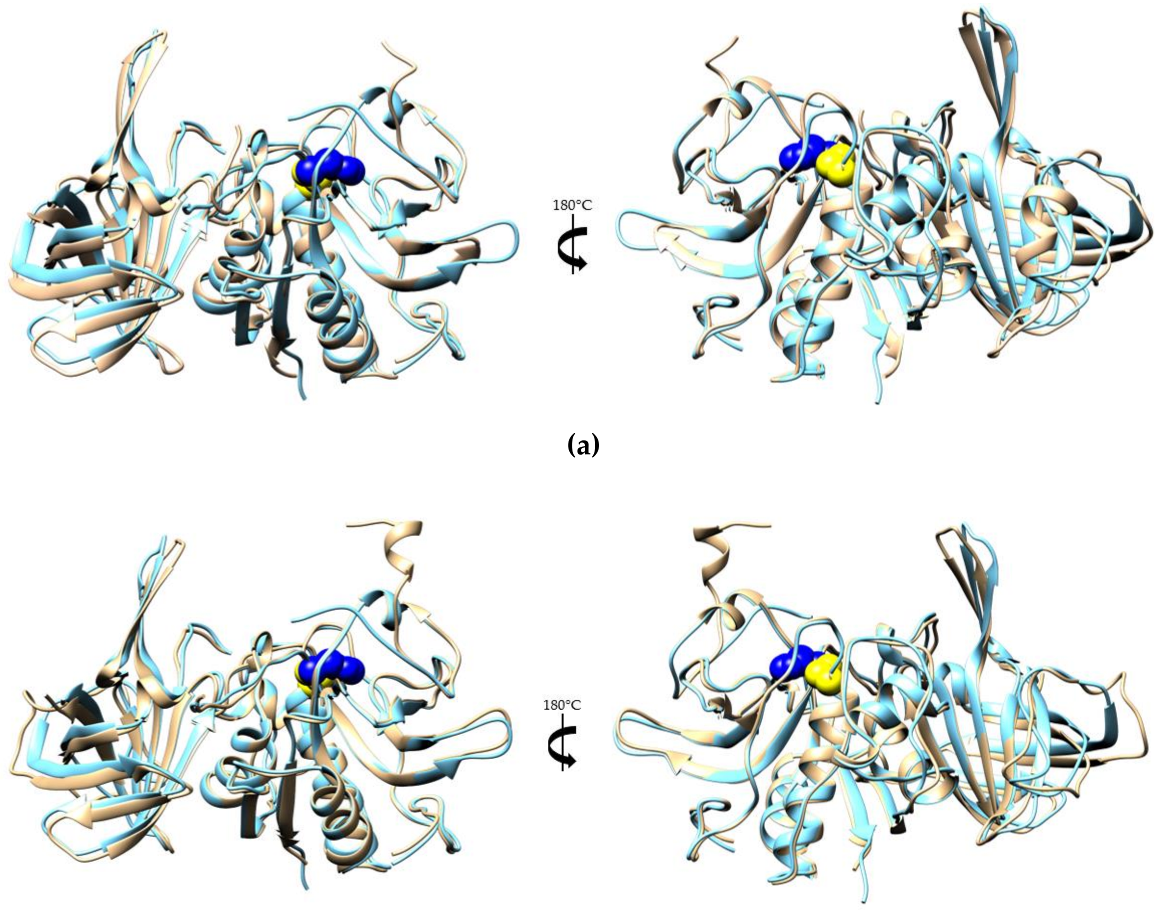

(a)

(b)

Supplementary Figure S3. Pairwise superposition of the crystal structure of human DPPI (shown in blue) with homology models of its DPPI(1) from *Naegleria gruberi* (a) and DPPI from *Ixodes ricinus* (b) (both shown in tan). The crystal structure was retrieved from the Protein Data Bank under accession number 1k3b. The homology models were built with AlphaFold, as described in the main text.

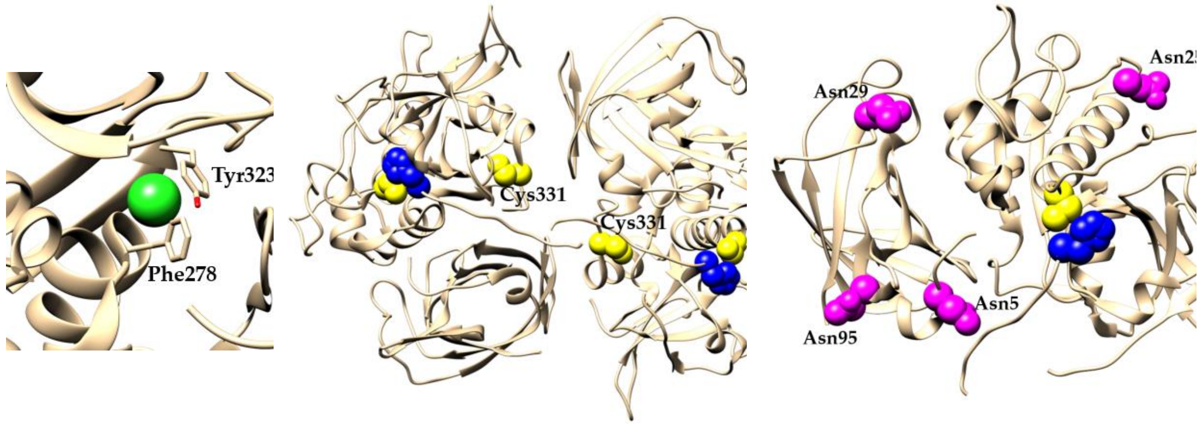

(**a**) (**b**) **(c)**

Supplementary Figure S4. Functionally important structural features shown in the crystal structure of human DPPI. (a) Chloride ion bound in the S2 binding pocket. The protein is shown in cartoon representation and the chloride ion as a sphere. Hydrophobic residues involved in chloride binding are shown as sticks and labelled. (b) Free Cys residue at position 331. A lateral dimer is shown in cartoon representation. Cys residues are shown as spheres. The catalytic diad is shown as spheres. (c) N-gylcosylation sites in human DPPI. A single subunit is shown in cartoon representation. Asn residues bearing N-linked glycans are shown as spheres. In all panels, residue numbering corresponds to the sequence of human proDPPI.

Supplementary Table S1. Accession numbers of sequences included in this work.

Name	Organism	Database	Accession number
DPPI	*Homo sapiens*	MEROPS	MER0001937
DPPI	*Pongo abelii*	MEROPS	MER0176577
DPPI	*Macaca fascicularis*	MEROPS	MER0055208
DPPI	*Bos taurus*	MEROPS	MER0031225
DPPI	*Canis lupus familiaris*	NCBI	NP_001182763.1
DPPI	*Rattus norvegicus*	MEROPS	MER0000705
DPPI	*Mus musculus*	MEROPS	MER0003458
DPPI	*Ornithorhynchus anatinus*	MEROPS	MER0097657
DPPI	*Crotalus adamanteus*	MEROPS	MER0363395
DPPI	*Anolis carolinensis*	MEROPS	MER0225683
DPPI	*Chelonia mydas*	UniProt	M7B428
DPPI	*Aligator mississippiensis*	MEROPS	MER0168135
DPPI	*Latimeria chalumnae*	MEROPS	MER0413848
DPPI	*Callorhinchus milli*	NCBI	XP_007900501.1
DPPI	*Cynoglossus semilaevis*	MEROPS	MER0567731
DPPI	*Danio rerio*	MEROPS	MER0054336
DPPI	*Astyanax mexicanus*	MEROPS	MER0365645
DPPI	*Xenopus tropicallis*	MEROPS	MER0065222
DPPI	*Xenopus laevis*	MEROPS	MER0036183
DPPI(1)	*Schistosoma japonicum*	NCBI	TNN09560.1
DPPI(2)	*Schistosoma japonicum*	NCBI	CAX71003.1
DPPI(1)	*Strongylocentrotus purpuratus*	MEROPS	MER0056650
DPPI(2)	*Strongylocentrotus purpuratus*	NCBI	XP_030853324.1
DPPI(1)	*Hydra vulgaris*	MEROPS	MER0159091
DPPI(2)	*Hydra vulgaris*	NCBI	XP_002160467.2
DPPI	*Ixodes ricinus*	MEROPS	MER0107071
DPPI	*Litopenaeus vannamei*	MEROPS	MER0158431
DPPI	*Daphnia pulex*	MEROPS	MER0344945
DPPI	*Frankliniella occidentalis*	UniProt	A0A6J1S139
DPPI	*Cryptotermes secundus*	UniProt	A0A2J7Q873
DPPI	*Trichoplax adhaerens*	MEROPS	MER0158665
DPPI	*Capitella capitata*	MEROPS	MER0254324
DPPI	*Saccoglossus kowalevskii*	MEROPS	MER0345012
DPPI(1)	*Aplysia californica*	MEROPS	MER0625643
DPPI(2)	*Aplysia californica*	NCBI	XP_012939419.1
DPPI	*Trichinella spiralis*	MEROPS	MER0345081
DPPI	*Capsaspora owczarzaki*	NCBI	XP_004345515.2
DPPI(1)	*Naegleria gruberi*	MEROPS	MER0345059
DPPI(2)	*Naegleria gruberi*	NCBI	XP_002674861.1
DPPI(3)	*Naegleria gruberi*	NCBI	XP_002670638.1
DPAP1	*Plasmodium falciparum*	UniProt	W7K503
CPC1	*Toxoplasma gondii*	UniProt	Q1AMF3
DPAP2	*Plasmodium falciparum*	UniProt	Q8I0V1
CPC2	*Toxoplasma gondii*	UniProt	A0A2T6IV78

continued from previous page

Name	Organism	Database	Accession number
DPAP3	*Plasmodium falciparum*	MEROPS	MER0078015
CPC3	*Toxoplasma gondii*	NCBI	XP_018636232.1
DPPI	*Stylonychia lemnae*	NCBI	CCKQ01007168.1
DPPI	*Polarella glacialis*	NCBI	CAE8718510.1
DPPI	*Symbiodinium natans*	NCBI	CAE7248497.1
DPPI(1)	*Tetrahymena thermophila*	NCBI	XP 001022323.2
DPPI(2)	*Tetrahymena thermophila*	NCBI	XP 001023356.1
DPPI(1)	*Paramecium tetraurelia*	NCBI	XP 001455775.1
DPPI(2)	*Paramecium tetraurelia*	NCBI	XP 001448556.1
DPPI(3)	*Paramecium tetraurelia*	NCBI	XP 001456639.1
DPPI(4)	*Paramecium tetraurelia*	NCBI	XP 001431306.1
DPPI(1)	*Giardia intestinalis*	NCBI	XP 001709718.1
DPPI(2)	*Giardia intestinalis*	NCBI	XP 001709706.1
Papain	*Carica papaya*	Uniprot	P00784

Supplementary Table S2. Residues responsible for type I functional divergence between Amorphea and alveolate DPPIs using the cut-off value of posterior probability P > 0.5. Residue identities and numbering apply to human proDPPI.

**Residue**	**Posterior probability (P)**
Cys6	*0.503566*
Val16	*0.802016*
Ser21	*0.500327*
Ser31	*0.987135*
Val32	*0.992759*
Pro35	*0.999998*
Leu47	*0.550134*
Ala50	*0.810740*
Asp52	*0.999967*
Gly55	*0.999911*
Asn56	*0.991296*
Glu69	*0.502461*
Tyr75	*0.964776*
Phe81	*0.707830*
Val102	*0.567100*
His103	*0.977148*
Trp110	*0.875903*
Lys138	*0.601897*
Asn149	*0.682688*
Phe150	0.827397
Trp161	*0.973389*
Ala163	*0.725387*
Tyr166	*0.777973*
Pro191	*0.548247*
Asp212	*0.789497*
Arg214	*0.801381*
Pro224	*0.928635*
Arg226	*0.997172*

**Residue**	**Posterior probability (P)**
Phe237	*0.974604*
Glu244	*0.990599*
Ile249	*0.599313*
Leu250	*0.800145*
Asn253	*0.655373*
Gln269	*0.894134*
Asp289	*0.508465*
Val293	*0.525592*
Glu295	*0.915187*
Pro299	*0.986471*
Tyr300	*0.517352*
Tyr317	*0.500632*
Ser318	*0.971011*
Glu320	*0.585234*
Tyr321	*0.743106*
Val324	*0.725051*
Asn332	*0.574335*
Leu335	*0.881997*
Asp355	*0.963004*
Phe373	*0.657548*
Asn374	*0.965088*
Phe376	*0.528842*
Val383	*0.531112*
Thr390	*0.967060*
Val401	*0.511334*
Gly408	*0.839495*
Ile417	*0.887758*
Glu423	*0.718728*
